# Synthetic Boosted Resampling Using Deep Generative Adversarial Networks: A Novel Approach to Improve Cancer Prediction from Imbalanced Datasets

**DOI:** 10.3390/cancers16234046

**Published:** 2024-12-02

**Authors:** Fatih Gurcan, Ahmet Soylu

**Affiliations:** 1 Department of Management Information Systems, Faculty of Economics and Administrative Sciences, Karadeniz Technical University, 61080 Trabzon, Turkey; 2 Department of Computer Science, Faculty of Information Technology and Electrical Engineering, Norwegian University of Science and Technology, 2815 Gjøvik, Norway

**Keywords:** class imbalance, Generative Adversarial Networks (GANs), cancer diagnosis and prognosis, resampling strategies, ROC AUC, bagging and boosting models

## Abstract

Background/Objectives: This study examines the effectiveness of different resampling methods and classifier models for handling imbalanced datasets, with a specific focus on critical healthcare applications such as cancer diagnosis and prognosis. Methods: To address the class imbalance issue, traditional sampling methods like SMOTE and ADASYN were replaced by Generative Adversarial Networks (GANs), which leverage deep neural network architectures to generate high-quality synthetic data. The study highlights the advantage of GANs in creating realistic, diverse, and homogeneous samples for the minority class, which plays a significant role in mitigating the diagnostic challenges posed by imbalanced data. Four types of classifiers, Boosting, Bagging, Linear, and Non-linear, were assessed to evaluate their performance using metrics such as accuracy, precision, recall, F1 score, and ROC AUC. Results: Baseline performance without resampling showed significant limitations, underscoring the need for resampling strategies. Using GAN-generated data notably improved the detection of minority instances and overall classification performance. The average ROC AUC value increased from baseline levels of approximately 0.8276 to over 0.9734, underscoring the effectiveness of GAN-based resampling in enhancing model performance and ensuring more balanced detection across classes. With GAN-based resampling, GradientBoosting classifier achieved a ROC AUC of 0.9890, the highest among all models, demonstrating the effectiveness of GAN-generated data in enhancing performance. Conclusions: The findings underscore that advanced models like Boosting and Bagging, when paired with effective resampling strategies such as GANs, are better suited for handling imbalanced datasets and improving predictive accuracy in healthcare applications.

## Simple Summary

This study explores various resampling methods and classifiers for imbalanced datasets, focusing on cancer diagnosis and prognosis. Traditional methods like SMOTE and ADASYN were replaced by GANs, which generate high-quality synthetic data to address class imbalance. The study evaluated Boosting, Bagging, Linear, and Non-linear classifiers using metrics like accuracy and ROC AUC. Baseline models struggled with imbalanced data, emphasizing the need for resampling. GAN-based resampling significantly improved detection of minority classes and overall performance, boosting the average ROC AUC from 0.8276 to over 0.9734. GradientBoosting achieved the highest ROC AUC of 0.9890, showing GANs’ effectiveness in enhancing predictive accuracy.

## 1. Introduction

The application of machine learning in healthcare has revolutionized the way medical data are analyzed, enhancing early diagnosis, prognosis, and treatment strategies for various diseases. Machine learning-based algorithms contribute significantly to cancer research by enhancing early detection, prognosis, and treatment processes [1,2]. With the capability to analyze large and complex datasets, these algorithms enable a better identification of molecular-level markers and risk factors for cancer [1,3]. Specifically, deep learning and other supervised learning models are employed in image analysis, genetic data processing, and predictive analysis of patient data, facilitating the early detection of cancer and the personalization of treatment options [4]. These technologies assist researchers and healthcare professionals in developing more precise and effective solutions, opening new avenues in the fight against cancer. In the realm of oncology, particularly breast cancer, the use of predictive models can be pivotal for early detection and accurate prognosis, thereby improving patient outcomes [5,6]. One of the primary challenges in employing machine learning for medical purposes is the issue of class imbalance within datasets. This is especially true in data related to breast cancer, where instances of positive diagnoses (representing the minority class) are often outnumbered by negative cases (representing the majority class) [7,8]. Such imbalances can compromise the performance of machine learning models, resulting in biased predictions that favor the majority class and fail to accurately identify critical minority cases.

Recent research in the field has demonstrated the growing importance of advanced resampling techniques and ensemble learning to tackle class imbalance in medical datasets [7,9,10]. Studies have shown that traditional oversampling methods like SMOTE and ADASYN, while effective to some degree, may not fully capture the complexity of real data distributions in medical contexts [9,11,12,13,14,15]. For instance, recent work integrating GANs with classification models has shown promising results in generating synthetic samples that closely resemble the minority class, enhancing model performance in imbalanced scenarios [11,16]. GAN-based approaches have been successfully applied in studies focusing on cancer detection and prognosis, where generating realistic minority samples significantly improved recall and overall classification metrics [17,18]. Additionally, research on ensemble methods, such as Boosting and Bagging, has reinforced their ability to enhance model robustness and predictive accuracy, particularly when combined with innovative resampling techniques [10,18,19,20].

In recent years, Generative Adversarial Networks (GANs) have emerged as a powerful tool for synthetic data generation due to their ability to create high-quality, realistic samples by leveraging deep learning architectures [21,22]. GANs consist of two competing neural networks—the generator and the discriminator—that work in tandem to produce data that closely mimic real examples. The potential of GANs in addressing class imbalance lies in their capability to generate diverse and complex data, providing more robust support for minority classes compared to traditional oversampling methods [11,23]. Ensemble methods, known for their ability to combine multiple models to enhance predictive accuracy, have shown promise in mitigating issues related to data imbalance [9,10,24,25]. Boosting algorithms, for instance, sequentially train weak learners to correct misclassifications, thereby improving overall performance, while Bagging enhances model stability by training models in parallel to reduce variance [20,25,26]. Linear models, despite their simplicity, can be effective when used as baselines, and Non-linear models offer the flexibility to capture intricate relationships within data, which is critical for recognizing minority class patterns [13,27].

Given the current context, this study highlights the potential of combining deep generative models with supervised ensemble learning approaches to achieve more balanced results in health analytics. It also encourages further exploration of hybrid techniques for critical health applications, such as breast cancer prediction. This empirical study aims to investigate not only the standalone effectiveness of GANs for synthetic data generation but also the integration of these advanced data augmentation methods with different types of classifiers. This study leverages data from the SEER (Surveillance, Epidemiology, and End Results) breast cancer database and incorporates GANs for synthetic data generation to address class imbalance. It also analyzes and compares the performance of four different types of supervised learning models: Boosting, Bagging, Linear, and Non-linear classifiers. These models were chosen for their varied approaches to learning and generalization. Metrics such as accuracy, precision, recall, F1 score, and ROC AUC are used to assess model performance, highlighting the ability of these methods to improve the recognition and diagnosis of cases within the minority class. Through a comprehensive analysis, this study revealed the comparative performance of these four classifier types when handling class-imbalanced data, and to underscore the potential benefits of combining GAN-based synthetic oversampling with advanced classification techniques. The findings offer valuable insights for the development of robust predictive models in the field of breast cancer research, contributing to more balanced and effective healthcare solutions.

## 2. Materials and Methods

**Figure 1:**
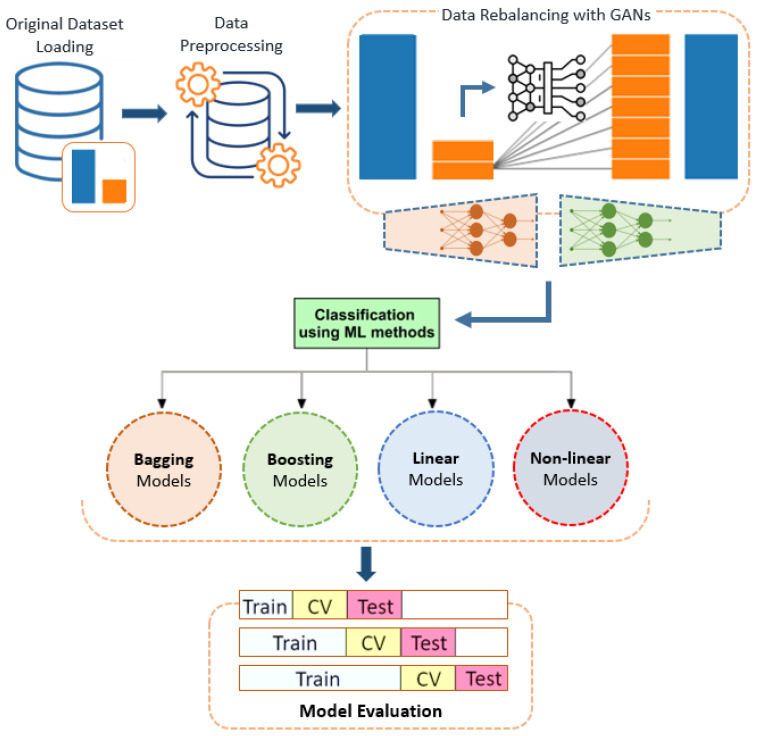
Flowchart of the study methodology.

This section outlines the materials and methodologies used in this study to evaluate the performance of various classification models and resampling techniques, with a particular focus on addressing class imbalance using Generative Adversarial Networks (GANs) in the context of breast cancer prediction. The methodology described in this study is further detailed in the flowchart provided in Figure 1, which illustrates the step-by-step process of evaluating classification models and resampling techniques. This flowchart visualizes the sequential steps, starting with data acquisition from the SEER breast cancer database, followed by preprocessing and balancing using GAN-based synthetic sample generation. It then outlines the implementation of various classifiers and the process of integrating GANs to address class imbalance. Additionally, the flowchart highlights the GAN training process, the evaluation of model performance using specific metrics, and the final compilation and interpretation of results. This visual representation serves to clarify the systematic approach used in the study and the interconnections between each step in the process. In the following sections, we will discuss these stages in detail, addressing each step in the process.

### 2.1. Data Description

The SEER Breast Cancer Dataset is a valuable resource for in-depth breast cancer prediction research, encompassing clinical, demographic, and pathological data on 4024 patients. It includes a range of 16 detailed variables that provide critical insights into patient profiles, disease characteristics, and treatment factors [28]. Key features include age, which influences treatment decisions, race and marital status, which capture demographic and social support factors impacting patient outcomes, and tumor stage (T Stage) and node stage (N Stage), which are essential for clinical cancer staging and reflect tumor size, spread, and lymph node involvement [28,29]. Furthermore, differentiation and grade assess the abnormality and aggressiveness of the tumor cells, while estrogen and progesterone status offer an insight into hormone receptor status, crucial for guiding hormone therapy treatment options. Additional features include tumor size, measured in millimeters, and regional node examined/positive, which detail the extent of diagnostic efforts and disease spread. the survival months variable records patient survival duration, central to understanding long-term outcomes. The target variable, Status, indicates survival outcome (“Alive” or “Dead”), with 84.4% of patients labeled as “Alive” and 15.6% as “Dead”, resulting in a substantial imbalance ratio (approximately 5.5) for class separation. This imbalance presents challenges for accurate model training, as models can become biased toward the majority class. Addressing this imbalance requires advanced resampling and modeling techniques to accurately predict survival outcomes for the minority class [14,29,30].

### 2.2. Data Acquisition and Preprocessing

The analysis began by acquiring the breast cancer dataset stored in CSV format, containing key attributes for predicting cancer status. After loading the data, preprocessing steps were undertaken to ensure compatibility with machine learning algorithms and maximize model performance [9,12,31,32,33]. One essential step was encoding categorical features into numerical values, a necessary transformation for algorithms that do not handle categorical data directly [34,35]. We applied LabelEncoder to all object-type columns, which converted categorical labels into integer representations without losing categorical relationships [32,34]. Subsequently, we separated the feature matrix X from the target variable status, which contains cancer diagnosis labels. To optimize the learning process, all feature values were standardized using StandardScaler, which adjusted the data to have a mean of zero and a unit variance [9,12,35]. Standardization aids convergence and accuracy during training, especially for distance-based algorithms, by removing scale disparities among features [9,36]. In this analysis, the dataset is divided into training and test sets using 10-fold stratified cross-validation, implemented with StratifiedKFold [8,30,32]. This approach splits the dataset into 10 equally sized folds, preserving the class distribution in each fold, and iteratively uses each fold as a test set while training on the remaining nine folds. This preprocessing provided a refined dataset that was fully numeric and normalized, ready for input into various machine learning models [20,30].

### 2.3. Addressing Class Imbalance with GANs

Breast cancer datasets often exhibit class imbalance, where benign samples significantly outnumber malignant ones. This imbalance can lead to biased model predictions, favoring the majority class while potentially missing minority class instances crucial to accurate cancer diagnosis [14,37,38]. To quantify this imbalance, we calculated the class distribution and imbalance ratio, the latter representing the ratio of the majority to the minority class [39]. A high imbalance ratio often necessitates resampling techniques, as was done here [9,10]. In addressing class imbalance for the experimental dataset, we selected Generative Adversarial Networks (GANs) over traditional resampling techniques due to GANs’ unique capability to generate synthetic samples that closely resemble real data [21,22,23]. While oversampling and undersampling methods such as SMOTE or random resampling can adjust class distributions, these methods risk either oversimplifying minority data patterns or discarding valuable information from the majority class [40,41].

GANs were selected for this study due to their demonstrated superiority in generating highly realistic and diverse synthetic samples, particularly in the context of imbalanced datasets. Unlike variational autoencoders (VAEs), which optimize for a balance between reconstruction fidelity and latent space regularization, GANs employ a dynamic adversarial framework that directly focuses on producing synthetic data indistinguishable from real samples [21,42]. This capability is especially crucial in healthcare datasets, where preserving the underlying distribution and variability of minority class data is vital for accurate prediction. Additionally, GANs are adept at modeling complex, high-dimensional data distributions, making them particularly well-suited for datasets like SEER, which include nuanced clinical and pathological features. While VAEs and other generative methods have their merits, prior studies have reported that GANs often outperform VAEs in producing visually and structurally consistent data, which aligns with the needs of this study [21,42,43].

GANs, on the other hand, leverage a dynamic learning process between a generator and a discriminator to produce highly realistic synthetic samples, preserving the complexity and structural characteristics of the original data [11,17,21]. The GAN architecture consisted of two neural networks: a generator, responsible for creating synthetic samples, and a discriminator, tasked with differentiating real samples from synthetic ones [11,17,23]. Together, these components enable the GAN to iteratively refine the generated samples to resemble actual minority data, improving the model’s ability to learn features from both classes and reducing the effects of imbalance [9,39]. The minority class data generated by the GAN were used to incrementally balance the imbalance ratio (IR) in five different scenarios, reducing it from 5.5 to 1. Each scenario involved a different level of oversampling, which was applied to progressively decrease the IR, aiming to achieve a more balanced dataset for model training [22,23]. Finally, both minority and majority classes were oversampled using GAN to generate additional synthetic data, and experiments were conducted with the goal of achieving an imbalance ratio (IR) of 1, further enhancing the dataset’s balance. This nuanced data generation helps models generalize better by exposing them to more diverse instances, thus improving their capacity to learn meaningful patterns from both classes without losing critical information [11,23].

### 2.4. GAN Training Process

**Figure 2:**
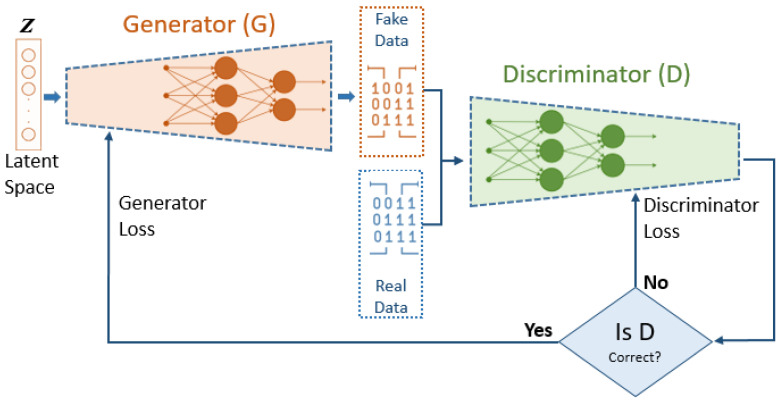
Flowchart of the GAN training process.

The training process of the generative adversarial network (GAN) follows an iterative adversarial framework that alternates between two main components: the generator and the discriminator. These two networks work in tandem, with each attempting to outperform the other through continuous competition [22,43]. Figure 2 illustrates the GAN training process, highlighting the key steps involved in optimizing both the generator and discriminator. The GAN employed consists of two primary components: the generator and the discriminator, both implemented as feedforward neural networks.

Generator Architecture: The generator takes a latent vector as input, with a dimensionality of 100. It consists of three dense layers:

- The first layer has 256 neurons with a ReLU activation function.
- The second layer consists of 128 neurons, also using a ReLU activation function.
- The final output layer has a number of neurons matching the input feature dimension of the dataset, with a sigmoid activation function to produce normalized synthetic data.

Discriminator Architecture: The discriminator is a binary classifier designed to distinguish real data from synthetic data. It consists of three dense layers:

- The input layer has a size equal to the number of input features.
- The first hidden layer contains 256 neurons with a ReLU activation function.
- The second hidden layer has 128 neurons, also using ReLU activation.
- The output layer has a single neuron with a sigmoid activation function to predict whether the input is real or generated.

Figure 2 emphasizes how the generator’s goal is to minimize its loss by producing increasingly realistic samples, while the discriminator aims to correctly classify real and synthetic data, minimizing its own loss. This flowchart demonstrates the adversarial dynamic between the generator and discriminator, where the losses of both networks evolve during training. As the generator’s loss decreases, it produces more convincing synthetic data, and the discriminator’s loss reflects its growing ability to differentiate between real and fake samples. The generator starts by taking a random noise vector, often sampled from a standard distribution, and transforms it into synthetic data that resemble the minority class in the dataset. Initially, the generated data are crude and unrealistic. The discriminator’s task is to differentiate between real data samples (from the actual minority class) and the synthetic data produced by the generator. It receives binary labels to identify whether each input is real or fake, allowing it to learn the distinguishing features of the genuine class distribution [21,43,44].

During each training epoch, the process is divided into two critical steps. First, the discriminator is updated by training it on both real data (from the actual minority class) and synthetic data (generated by the generator) [11,17,21]. The discriminator learns to classify these samples, improving its ability to distinguish real instances from the generated ones. This feedback allows the discriminator to become increasingly adept at identifying synthetic samples. In the second step, the generator is trained to improve its data generation capabilities by optimizing its parameters to “fool” the discriminator into classifying its synthetic samples as real [11,17]. This is done by minimizing the loss function that penalizes the generator when its output is incorrectly classified as fake by the discriminator. The generator receives the gradient from this loss, which guides it in refining its output to appear more realistic in subsequent epochs [21,43].

**Figure 3:**
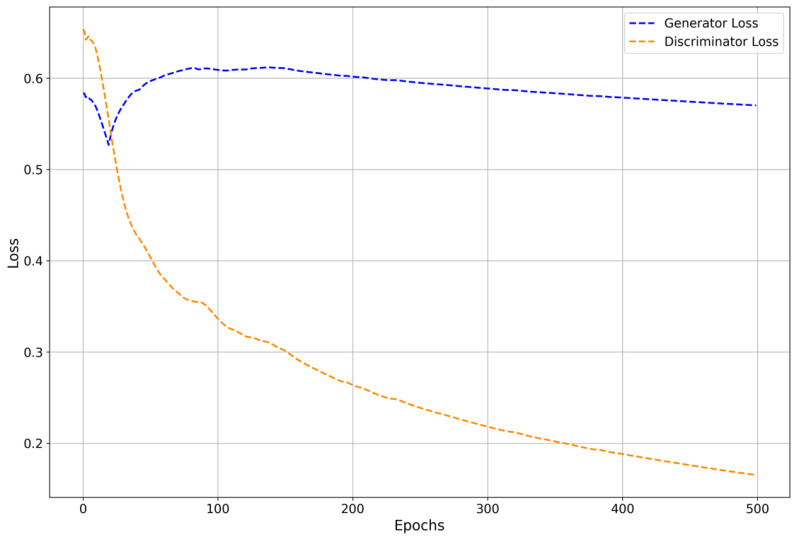
Generator and discriminator losses for the GAN training process.

The training continues for multiple epochs, with both networks gradually improving. The generator refines its ability to produce synthetic data that become more similar to the real data, while the discriminator sharpens its ability to distinguish between real and synthetic instances [17,22,23]. This dynamic adversarial training process helps both components evolve in a complementary manner. The generator produces higher-quality synthetic samples as training progresses, while the discriminator gains better accuracy in identifying subtle differences between real and synthetic data [17,23,43]. Figure 3 illustrates the loss curves for both the generator and discriminator during the GAN training process, showing the adversarial dynamics as they evolve over epochs. This figure highlights the gradual improvement in the generator’s ability to create realistic samples, while the discriminator becomes more adept at distinguishing between real and synthetic data as training progresses.

By the end of the training process, the generator is capable of producing highly realistic synthetic data that closely resemble the minority class in terms of structure and distribution [21,23]. These synthetic samples can be added to the minority class in the dataset, effectively balancing the class distribution and addressing the class imbalance issue [11,44]. The improved dataset, now enriched with realistic minority class samples, can then be used to train downstream classification models. The high-quality synthetic samples generated by the GAN not only help balance the dataset but also enable the models to learn more meaningful patterns from both the majority and minority classes, thus improving the overall performance and robustness of the classifiers.

### 2.5. Classification Models and Evaluation

In this analysis, we employed four distinct types of supervised classifiers: Bagging, Boosting, Linear, and Non-linear models, each bringing unique advantages to the classification task [19,20,26,45,46]. Bagging ensemble techniques, such as the Bagging classifier with RandomForest as the base estimator, help improve model stability and accuracy by reducing variance through aggregating predictions from multiple trees [9,24,27]. The RandomForest and ExtraTrees classifiers further exemplify this approach, utilizing ensembles of decision trees to enhance predictive performance. Boosting methods, including GradientBoosting, XGBoost, and CatBoost, focus on sequentially training models, where each new model aims to correct errors made by the previous ones. This technique effectively increases model accuracy, particularly in handling complex datasets with class imbalance [32,34,35,47].

For linear classifiers, we utilized LogisticRegression for its straightforward interpretation and effectiveness in binary classification contexts [27,48,49]. The LinearSVC employs a linear kernel to maximize the margin between classes, while PassiveAC (PassiveAggressiveClassifier), used in conjunction with CalibratedClassifierCV, provides a robust approach to improving probability estimates [12,47,50]. Lastly, we explored Non-linear models, such as KNeighbors and SVC, which are adept at capturing intricate relationships within the data [3,49]. The DecisionTree rounds out this category, offering a transparent decision-making process by splitting data based on feature values [51]. To evaluate the performance of these diverse classifiers on the balanced dataset, we implemented Stratified K-Fold cross-validation, which divides the dataset into ten folds while preserving the class distribution in each fold [8,9,30]. This systematic approach ensures that each classifier is exposed to representative samples from the balanced dataset, minimizing biases from uneven splits [8,30].

### 2.6. Performance Assessment, and Results Compilation

Following model training, performance metrics were calculated to gauge each classifier’s effectiveness, incorporating the benefits of Stratified K-Fold cross-validation in the assessment process [8,30,47]. Key metrics included accuracy, F1 score, precision, recall, and ROC AUC, each providing unique insights into the model’s handling of the balanced dataset [9,52,53]. The use of Stratified K-Fold ensures that each fold maintains the same proportion of classes as the original dataset, which is particularly advantageous in imbalanced contexts. This stratification reduces variance in the evaluation process, leading to more reliable and generalizable performance metrics [47,54]. Accuracy and F1 score provided overall performance metrics, with the F1 score specifically balancing precision and recall, making it especially useful in situations where class distributions are uneven. Precision indicates the proportion of true positive results among all positive predictions, while recall reflects the ability of the classifier to identify all relevant instances of the minority class [52,54]. ROC AUC was included to assess each classifier’s ability to discriminate between classes, with a higher ROC AUC indicating better discrimination and robustness against false positives [45,52]. Results from each model and each configuration of latent dimensions and batch sizes were stored in a structured DataFrame for in-depth analysis and comparison. This thorough performance assessment offered insights into the optimal classifiers and configurations for balanced breast cancer classification, demonstrating the benefits of GAN-based resampling in handling imbalanced medical datasets [9,11,45,53].

## 3. Results and Discussion

### 3.1. Scenario 1: Baseline Scenario at an Imbalance Ratio of 5.5 Without Resampling

The baseline scenario, with an imbalance ratio of 5.5, highlights the strengths and limitations of different classifiers when applied without resampling, revealing key insights into their classification capabilities. The significant class imbalance poses challenges for many models, as it can lead to biased performance skewed towards the majority class. Table 1 presents the performance of classifiers in the baseline scenario without resampling, sorted in descending order according to their ROC AUC scores. The results show that GradientBoosting achieved the highest ROC AUC score (0.8667), indicating its superior ability to distinguish between classes under these conditions. Following closely, CatBoost (0.8628) and LogisticRegression (0.8626) also demonstrated commendable ROC AUC values, suggesting these boosting and linear classifiers perform relatively well despite the class imbalance.

LinearSVC and Bagging classifiers had slightly lower ROC AUC scores at 0.8605 and 0.8583, respectively, showing their potential but highlighting the impact of imbalanced data on their recall, as reflected in their F1 scores. Notably, RandomForest had a moderate ROC AUC of 0.8483, indicating a decent balance between true positive and false positive rates but struggling with recall, similar to other models. Lower down the list, ExtraTrees (0.8394) and XGBoost (0.8298) displayed lower ROC AUC scores, pointing to more significant challenges in handling class imbalance effectively. Non-linear models such as SVC and KNeighbors exhibited more pronounced difficulties, with ROC AUCs of 0.8215 and 0.7784, respectively, showing weaker performance in distinguishing between classes. The PassiveAC and DecisionTree classifiers had the lowest ROC AUC scores of 0.8031 and 0.6994, indicating substantial limitations in handling the imbalanced dataset, with DecisionTree showing the most pronounced struggle in achieving reliable classification metrics.

Overall, Table 1 underscores that boosting methods generally perform better in handling imbalanced scenarios, yet Recall remains an area requiring significant improvement. Notably, most classifiers show ROC AUC scores below 0.87, signaling a clear need for further optimization to achieve more robust performance. This highlights the importance of implementing targeted strategies such as resampling or model tuning to enhance the classifiers’ ability to manage class imbalance effectively. Specifically, applying techniques like GANs (Generative Adversarial Networks) for synthetic data generation could provide a significant contribution by boosting the minority class representation.

### 3.2. Scenario 2: Minority Oversampling Scenario with an Imbalance Ratio of 3

In this scenario, the minority class count was increased from 616 to 1136 to address the class imbalance. In the first instance, applying minority oversampling with GANs reduced the imbalance ratio from 5.5 to 3, leading to significant improvements in classifier performance across various metrics. Table 2 presents the performance of classifiers after applying minority oversampling. Table 2 presents the performance of classifiers after applying minority oversampling, ranked in descending order based on the last column, ROC AUC. In this scenario, where minority oversampling was used to reduce the imbalance ratio to 3, GradientBoosting stands out as the top-performing classifier with a ROC AUC of 0.9276, indicating its strong ability to differentiate between classes. CatBoost follows closely with a ROC AUC of 0.9244, showcasing its robustness in handling class imbalance effectively. Bagging and RandomForest also perform well, achieving ROC AUC scores of 0.9216 and 0.9174, respectively, emphasizing the effectiveness of bagging methods in enhancing classification performance under these conditions.

ExtraTrees demonstrates a solid performance with a ROC AUC of 0.9134, showing it is still a reliable model in managing imbalanced data after oversampling. XGBoost follows with a ROC AUC of 0.9111, reinforcing the general effectiveness of boosting techniques in complex classification tasks. Among Non-linear models, SVC achieves a notable ROC AUC of 0.8990, showing competitive results compared to ensemble methods, while KNeighbors trails with a ROC AUC of 0.8722. Linear models such as LogisticRegression and LinearSVC display moderate performance with ROC AUC scores of 0.8407 and 0.8402, respectively, indicating that although they are dependable, they may not match the precision of ensemble techniques under oversampled conditions. DecisionTree shows a lower ROC AUC of 0.8131, suggesting it is less suited for scenarios with moderate class imbalance. PassiveAC performs the lowest, with a ROC AUC of 0.8043, highlighting its limited ability to handle imbalanced data effectively. Overall, Table 2 underscores that ensemble methods, particularly boosting and bagging classifiers, tend to achieve the highest ROC AUC scores in minority oversampling scenarios, demonstrating their suitability for tasks requiring robust class discrimination.

### 3.3. Scenario 3: Minority Oversampling Scenario with an Imbalance Ratio of 2

In this scenario, where minority oversampling is applied and the imbalance ratio is reduced to 2, the minority class count increased from 616 to 1704, leading to significant improvements in classifier performance across multiple evaluation metrics. This improvement is particularly notable compared to both the baseline scenario and the previous oversampling with imbalance ratios of 5.5 and 3. Table 3 demonstrates the positive impact of reducing the imbalance ratio to 2 through minority oversampling. The classifiers, especially boosting and bagging methods, have been able to better capture the minority class while maintaining high precision, recall, and F1 scores. Furthermore, the classifiers in Table 3 are ranked according to the ROC AUC score, further highlighting the effectiveness of oversampling in handling class imbalance in classification tasks. The classifiers, particularly those using boosting methods, show a marked increase in accuracy, precision, recall, and F1 scores.

GradientBoosting leads with the highest ROC AUC score of 0.9538, indicating its superior performance in distinguishing between classes, followed closely by CatBoost with a score of 0.9493. These boosting methods clearly outperform the rest of the classifiers in terms of ROC AUC, highlighting their effectiveness in capturing both the majority and minority classes. Bagging and RandomForest follow with ROC AUC values of 0.9485 and 0.9462, respectively, showing that bagging methods also provide strong discriminatory power. XGBoost, another boosting method, comes in with a ROC AUC of 0.9391, still demonstrating solid performance, though slightly behind the top performers.

In comparison, SVC and KNeighbors, while excelling in precision, have lower ROC AUC scores (0.9321 and 0.9168, respectively), suggesting that while they may be good at identifying positive instances, they struggle more with the overall class distinction compared to boosting and bagging methods. Linear models such as LogisticRegression, LinearSVC, and PassiveAC show comparatively lower ROC AUC values, with LogisticRegression and LinearSVC performing similarly at 0.8946 and 0.8915, while PassiveAC has the lowest ROC AUC at 0.8858. Overall, Table 3 demonstrates how classifiers perform when ranked by ROC AUC, emphasizing the significant advantages of boosting and bagging methods in reducing class imbalance and improving model effectiveness. The ROC AUC scores further reinforce the impact of reducing the imbalance ratio to 2 through minority oversampling, as these models exhibit improved classification capabilities.

### 3.4. Scenario 4: Minority Oversampling Scenario with an Imbalance Ratio of 1

In this scenario, only minority class sample sizes were increased from 616 to 3408, the Imbalance Ratio (IR) is reduced to 1 through minority oversampling using GANs. In Table 4, the classifiers are again ranked primarily by their ROC AUC values, reflecting their overall classification performance, especially in distinguishing between the minority and majority classes. GradientBoosting leads with the highest ROC AUC score of 0.9764, followed by Bagging with 0.9734. These two classifiers dominate in terms of ROC AUC, demonstrating their strong ability to effectively classify both classes in a balanced dataset with an Imbalance Ratio of 1. CatBoost and RandomForest also show impressive ROC AUC scores of 0.9733 and 0.9721, respectively, indicating that boosting and bagging methods continue to perform well even as the class distribution approaches balance. ExtraTrees, with a ROC AUC score of 0.9698, maintains strong discriminatory performance, although it falls slightly behind the top performers in this table. XGBoost exhibits a ROC AUC of 0.9695, which is comparable to ExtraTrees, further supporting the efficacy of boosting methods.

SVC, while offering the highest precision (0.9853), has a ROC AUC score of 0.9637, indicating that although it is highly precise, it is slightly less effective in distinguishing between the classes compared to the top boosting and bagging methods. KNeighbors also performs well with a ROC AUC of 0.9558, showing that Non-linear models can still achieve strong results with reduced class imbalance. Linear models like PassiveAC, LinearSVC, and LogisticRegression exhibit lower ROC AUC values, with PassiveAC scoring the lowest at 0.9423, though it still performs adequately in distinguishing the classes. Lastly, DecisionTree has the lowest ROC AUC score of 0.9033, suggesting that, while effective in some cases, it struggles more with distinguishing between the minority and majority classes compared to other classifiers.

Overall, Table 4 highlights the continued dominance of boosting and bagging methods in achieving the highest ROC AUC values. This demonstrates the effectiveness of these models in handling class imbalance even when the Imbalance Ratio is reduced to 1. Despite the overall improvement in model performance as the class distribution becomes more balanced, boosting and bagging classifiers still outperform the others in their ability to differentiate between classes.

### 3.5. Scenario 5: Majority and Minority Oversampling Scenario with an Imbalance Ratio of 1

In this scenario, both the majority and minority class sample sizes were increased to 5000 using GANs, achieving balanced data through both majority and minority oversampling. Table 5 illustrates the performance of various classifiers ranked by their ROC AUC scores. GradientBoosting leads with the highest ROC AUC of 0.9890, showcasing its superior capability in distinguishing between classes. This is closely followed by Bagging, which also performs exceptionally well with a ROC AUC of 0.9880, emphasizing the strength of ensemble methods in handling class imbalance effectively. CatBoost continues to be a top performer with a ROC AUC of 0.9870, highlighting its robust classification capabilities when both majority and minority oversampling techniques are employed. RandomForest achieves a ROC AUC of 0.9869, demonstrating its strong predictive power in balanced data scenarios. ExtraTrees also delivers solid performance with a ROC AUC of 0.9859, proving effective in leveraging the full potential of bagging for classification tasks.

XGBoost follows with a ROC AUC of 0.9857, underscoring the reliability of boosting algorithms in achieving high-classification metrics. Among Non-linear models, KNeighbors achieves a notable ROC AUC of 0.9756, while SVC has a strong showing at 0.9744, indicating that these models can still achieve competitive results when the class distribution is balanced. PassiveAC, LogisticRegression, and LinearSVC deliver moderate ROC AUC scores of 0.9634, 0.9569, and 0.9563, respectively, indicating their reliable, though slightly lower, discriminatory power compared to ensemble methods. Finally, DecisionTree has the lowest ROC AUC of 0.9312 in this scenario, reflecting its relatively limited performance when compared to more advanced ensemble and boosting methods.

Overall, the results in Table 5 demonstrate that reducing the Imbalance Ratio to 1 through majority and minority oversampling enhances the performance of most classifiers, with boosting and bagging methods consistently achieving the highest ROC AUC scores. This indicates that ensemble techniques remain highly effective for classification tasks in balanced data settings.

## 4. Conclusions

This study has evaluated the performance of different sampling methods and classifier models in imbalanced datasets, particularly in critical healthcare areas such as cancer diagnosis and prognosis, and investigated the effectiveness of various strategies. To address the class imbalance problem, traditional sampling methods like SMOTE and ADASYN were replaced with synthetic data generation methods based on GANs (Generative Adversarial Networks), designed with deep artificial neural network architecture. GANs have drawn attention with their ability to generate high-quality data, especially in correcting imbalanced class distributions with high-quality data samples. According to our findings, the superior data generation capacity offered by GANs has enabled the creation of more realistic, homogeneous, and diversified examples of the minority class, thus making an effective contribution to overcoming the diagnostic and prognostic challenges caused by class imbalance.

In terms of classifier types, four different types of classifiers, including Boosting, Bagging, Non-linear, and Linear classifiers, were used in this study, and the performance of each model was evaluated using metrics such as accuracy, F1 score, recall, and ROC AUC. The preference for Bagging and Boosting is based on the potential of these models to better handle the issues arising from data imbalance. Bagging, in particular, reduces generalization error by increasing model diversity, while Boosting improves classification accuracy by continuously correcting misclassified examples. These features are reflected in higher accuracy and ROC AUC scores, particularly for the minority class. Linear and Non-linear classifiers were added to observe the effects of simpler models when combined with GANs’ boosting effects. Linear models, with their simpler structure and faster processing, sometimes produce good results, while Non-linear models have the capacity to learn more complex data structures and relationships, which can be particularly useful for recognizing the minority class in imbalanced datasets.

Our study shows that, besides data generation with GANs, Bagging and Boosting models effectively handled class imbalance and improved model performance in critical health issues like cancer. In high-class imbalance scenarios (imbalance ratio: 5.5), Boosting methods achieved higher accuracy and better F1 scores, but recall values remained low, indicating difficulty in recognizing the minority class. Despite high accuracy in models like GradientBoosting and CatBoost, their low recall emphasizes the need for additional strategies to improve performance in imbalanced datasets. After applying oversampling to the minority class, significant improvements in recall and ROC AUC values were observed, particularly for GradientBoosting and CatBoost models, which showed over 90% improvement. This enhancement boosted the model’s accuracy and helped balance the class distribution. When both majority and minority oversampling (imbalance ratio: 1) were applied, boosting and bagging models, including GradientBoosting and Bagging, achieved high accuracy and ROC AUC scores above 0.96. These results demonstrate that more complex boosting and bagging models perform better in imbalanced datasets.

This study focused on demonstrating the feasibility of GAN-based resampling for addressing class imbalance, but several important considerations remain unaddressed. A key limitation is the use of a basic GAN without directly comparing its performance to advanced models, such as conditional GANs (cGANs). While basic GANs model joint probability distributions to generate diverse synthetic samples, cGANs incorporate class labels to produce more targeted and potentially higher-quality samples for the minority class. This theoretical advantage has not been empirically tested in this study. A direct comparison between basic GANs and cGANs could provide deeper insights into the benefits of conditional probability modeling for improving classification in imbalanced datasets. Additionally, exploring other advanced GAN models, such as CycleGAN or BigGAN, in combination with ensemble methods, could further enhance synthetic data generation. For instance, CycleGAN could be employed to map relationships between minority and majority class distributions, potentially improving the overall ability of classifiers to handle imbalanced data. Self-Training GANs (ST-GANs) offer another avenue for improvement, as they iteratively refine synthetic samples based on the predictions of an initial classifier, ensuring that the generated data closely resemble real-world samples.

The study’s findings are also limited by the absence of external validation on independent datasets, which may constrain the generalizability of the results. Future research should validate the proposed GAN-based resampling method using datasets from diverse sources and populations to ensure its robustness across various contexts and cancer types. Lastly, the computational demands of GAN training, particularly for large datasets, pose challenges in resource-constrained environments. Addressing these challenges could involve employing pre-trained models or adopting more efficient architectures like Wasserstein GANs (WGANs) and cGANs to reduce training times and resource requirements. Techniques such as model pruning and distributed training could further optimize the performance and feasibility of these methods for real-time clinical applications.

Incorporating adversarial training into ensemble models, where a generator is trained alongside the classifier in an adversarial setting, could enhance model robustness to overfitting and improve performance on imbalanced datasets. However, GANs face limitations such as mode collapse, overfitting, and representational biases, which may affect the diversity and quality of synthetic data. Ensemble methods, while effective, can also struggle with overfitting and bias toward the majority class in highly imbalanced scenarios. Exploring GAN-based synthetic data generation integrated with deep learning ensemble methods, such as Deep Neural Networks (DNNs), Convolutional Neural Networks (CNNs), or Recurrent Neural Networks (RNNs), could further enhance classification performance by capturing more complex representations of minority class features.

Additionally, combining GANs with advanced deep learning ensembles like Deep Forest or DeepBoost presents a promising direction. For instance, Deep Forest, a tree-based ensemble leveraging deep learning, could benefit from GAN-generated synthetic data to better detect minority class instances. Stacked DNNs, where multiple deep models are layered and their predictions combined, could also improve classification performance by incorporating diverse synthetic samples. These approaches offer the potential to tackle class imbalance in complex healthcare datasets more effectively. In conclusion, while this study shows potential in addressing class imbalance, its practical application in healthcare systems warrants further exploration. Integrating GAN-based resampling into clinical workflows, particularly for predictive models in cancer diagnosis, could enhance real-time decision-making. Addressing challenges such as data processing speed and computational efficiency will be key to improving patient outcomes and operational feasibility in healthcare settings.

## Figures and Tables

**Table 1:** Baseline scenario performance with imbalance ratio of 5.5 without resampling.

Classifier	Type	Accuracy	Precision	Recall	F1 Score	ROC AUC
GradientBoosting	Boosting	0.9068	0.8573	0.5262	0.6327	0.8667
CatBoost	Boosting	0.9046	0.8566	0.5051	0.6175	0.8628
LogisticRegression	Linear	0.8954	0.8406	0.4484	0.5642	0.8626
LinearSVC	Linear	0.8926	0.8441	0.4093	0.5353	0.8605
Bagging	Bagging	0.9073	0.8735	0.4938	0.6181	0.8583
RandomForest	Bagging	0.9043	0.8616	0.4905	0.6086	0.8483
ExtraTrees	Bagging	0.8897	0.8300	0.4093	0.5294	0.8394
XGBoost	Boosting	0.8954	0.8240	0.4986	0.5925	0.8298
SVC	Non-linear	0.8892	0.8560	0.3541	0.4921	0.8215
PassiveAC	Linear	0.8551	0.8670	0.0695	0.1208	0.8031
KNeighbors	Non-linear	0.8760	0.7897	0.3540	0.4621	0.7784
DecisionTree	Non-linear	0.8332	0.6841	0.5065	0.4804	0.6994

**Table 2:** Classifier performance with minority oversampling at an imbalance ratio of 3.

Classifier	Type	Accuracy	Precision	Recall	F1 Score	ROC AUC
GradientBoosting	Boosting	0.9158	0.9039	0.7424	0.8143	0.9276
CatBoost	Boosting	0.9153	0.9133	0.7309	0.8113	0.9244
Bagging	Bagging	0.9138	0.9187	0.7186	0.8056	0.9216
RandomForest	Bagging	0.9136	0.9161	0.7204	0.8057	0.9174
ExtraTrees	Bagging	0.8984	0.9056	0.6641	0.7646	0.9134
XGBoost	Boosting	0.9081	0.8816	0.7309	0.7982	0.9111
SVC	Non-linear	0.8997	0.9390	0.6403	0.7608	0.8990
KNeighbors	Non-linear	0.8896	0.8845	0.6429	0.7438	0.8722
LogisticRegression	Linear	0.7576	0.5282	0.2867	0.3709	0.8407
LinearSVC	Linear	0.7794	0.6652	0.2410	0.3523	0.8402
DecisionTree	Non-linear	0.8511	0.6899	0.7371	0.7119	0.8131
PassiveAC	Linear	0.7616	0.6024	0.1346	0.2165	0.8043

**Table 3:** Classifier performance with minority oversampling at an imbalance ratio of 2.

Classifier	Type	Accuracy	Precision	Recall	F1 Score	ROC AUC
GradientBoosting	Boosting	0.9253	0.9384	0.8304	0.8809	0.9538
CatBoost	Boosting	0.9251	0.9466	0.8216	0.8795	0.9493
Bagging	Bagging	0.9263	0.9573	0.8152	0.8802	0.9485
RandomForest	Bagging	0.9257	0.9524	0.8181	0.8798	0.9462
ExtraTrees	Bagging	0.9102	0.9470	0.7747	0.8516	0.9432
XGBoost	Boosting	0.9173	0.9214	0.8222	0.8687	0.9391
SVC	Non-linear	0.9110	0.9659	0.7600	0.8500	0.9321
KNeighbors	Non-linear	0.9032	0.9396	0.7588	0.8389	0.9168
LogisticRegression	Linear	0.8652	0.7864	0.8181	0.8016	0.8946
LinearSVC	Linear	0.8641	0.7829	0.8198	0.8006	0.8915
PassiveAC	Linear	0.8686	0.8360	0.7558	0.7928	0.8858
DecisionTree	Non-linear	0.8746	0.7999	0.8334	0.8158	0.8643

**Table 4:** Classifier performance with minority oversampling at a 1:1 ratio.

Classifier	Type	Accuracy	Precision	Recall	F1 Score	ROC AUC
GradientBoosting	Boosting	0.9425	0.9722	0.9111	0.9406	0.9764
Bagging	Bagging	0.9449	0.9789	0.9094	0.9428	0.9734
CatBoost	Boosting	0.9428	0.9737	0.9102	0.9409	0.9733
RandomForest	Bagging	0.9434	0.9770	0.9082	0.9413	0.9721
ExtraTrees	Bagging	0.9328	0.9752	0.8882	0.9296	0.9698
XGBoost	Boosting	0.9383	0.9645	0.9099	0.9364	0.9695
SVC	Non-linear	0.9341	0.9853	0.8815	0.9304	0.9637
KNeighbors	Non-linear	0.9277	0.9729	0.8800	0.9240	0.9558
PassiveAC	Linear	0.9045	0.9110	0.8973	0.9037	0.9423
LinearSVC	Linear	0.9212	0.9336	0.9070	0.9201	0.9420
LogisticRegression	Linear	0.9127	0.9110	0.9149	0.9129	0.9415
DecisionTree	Non-linear	0.9033	0.8944	0.9149	0.9045	0.9033

**Table 5:** Classifier performance with majority and minority oversampling at a 1:1 ratio.

Classifier	Type	Accuracy	Precision	Recall	F1 Score	ROC AUC
GradientBoosting	Boosting	0.9611	0.9812	0.9402	0.9602	0.9890
Bagging	Bagging	0.9610	0.9847	0.9366	0.9600	0.9880
CatBoost	Boosting	0.9603	0.9810	0.9388	0.9594	0.9870
RandomForest	Bagging	0.9617	0.9845	0.9382	0.9608	0.9869
ExtraTrees	Bagging	0.9541	0.9834	0.9238	0.9526	0.9859
XGBoost	Boosting	0.9568	0.9739	0.9388	0.9560	0.9857
KNeighbors	Non-linear	0.9512	0.9808	0.9204	0.9496	0.9756
SVC	Non-linear	0.9549	0.9892	0.9198	0.9532	0.9744
PassiveAC	Linear	0.9268	0.9200	0.9354	0.9275	0.9634
LogisticRegression	Linear	0.9371	0.9312	0.9440	0.9375	0.9569
LinearSVC	Linear	0.9442	0.9473	0.9408	0.9440	0.9563
DecisionTree	Non-linear	0.9312	0.9283	0.9348	0.9314	0.9312

## Data Availability

The datasets used in this research are publicly available, and access links are provided as references in the dataset section of the article.
